# Comparative Effectiveness of Ultrasound‐Guided Corticosteroid Injection, Radiofrequency Ablation, and Their Combination for Recalcitrant Plantar Fasciitis: A Retrospective Cohort Study

**DOI:** 10.1002/jfa2.70080

**Published:** 2025-09-04

**Authors:** Çile Aktan, Cemil Aktan

**Affiliations:** ^1^ Department of Pain Medicine Antalya Training and Research Hospital Antalya Türkiye; ^2^ Department of Orthopedics and Traumatology Antalya Training and Research Hospital Antalya Türkiye

**Keywords:** corticosteroid injection, heel pain, pain management, plantar fasciitis, radiofrequency ablation, relapse, ultrasound‐guided therapy

## Abstract

**Background:**

Recalcitrant plantar fasciitis (PF) refers to persistent heel pain lasting ≥ 6 months despite appropriate conservative management, including physical therapy, orthotics, and pharmacological interventions. This study aimed to compare the clinical efficacy and safety of corticosteroid injection (CI), radiofrequency ablation (RFA), and their combination in patients with recalcitrant PF.

**Methods:**

In this retrospective study, a total of 156 patients with ultrasonographically confirmed plantar fasciitis, experiencing heel pain for at least 6 months and unresponsive to ≥ 3 months of standard conservative therapy, were included; 52 received RFA, 50 received CI, and 54 underwent combined therapy. Pain intensity (visual analog scale [VAS]), functional status (Foot Function Index [FFI], Roles and Maudsley score [RMS]), plantar fascia thickness (PFT), and relapse rates at 12 months were assessed. Within‐group and between‐group differences were assessed using appropriate nonparametric tests, and relapse rates were compared accordingly.

**Results:**

All treatment modalities improved VAS, FFI, RMS, and PFT at 6 months (*p* < 0.001). VAS declined from 6.73 to 6.81 at baseline to 1.62 in the RFA group and 1.83 in the combined group, whereas remaining at 6.56 in the CI group. FFI dropped from ∼52 to 21.50 and 17.57 in the RFA and combined groups but remained at 46.62 in the CI. PFT decreased from ∼6.2 mm to 3.29, 2.71, and 2.95 mm, respectively. Relapse occurred in 12 (23.1%), 19 (38.0%), and 8 (14.8%) patients in the RFA, CI, and combined groups. Between‐group differences were significant at 6 months (*p* < 0.001). No major adverse events were observed.

**Conclusion:**

Both CI and RFA are effective in recalcitrant PF, but their combination provides superior and more durable improvements in pain, function, and fascia morphology, with the lowest relapse rates. Ultrasound‐guided combined therapy suggests a safe, practical, and effective treatment option for patients unresponsive to conservative measures.

**Abbreviations**
CI: corticosteroid injection
FFI: Foot Function Index
PF: plantar fasciitis
PFT: plantar fascia thickness
RFA: radiofrequency ablation
RMS: Roles and Maudsley score
VAS: visual analog scale

## 1 Introduction

Plantar fasciitis (PF) is a leading cause of chronic heel pain, with a lifetime prevalence of 10%–15%, and accounts for over one million outpatient visits annually [1, 2]. Although conservative treatments, such as stretching and orthotics, are effective in most cases, approximately 10% of patients develop chronic recalcitrant PF, characterized by degenerative changes at the plantar fascia origin and persistent symptoms lasting more than 6 months [3, 4, 5].

Corticosteroid injection (CI) is a commonly used intervention due to its anti‐inflammatory properties and rapid pain relief. However, repeated injections offer diminishing returns and carry risks such as fat pad atrophy and fascial rupture [6, 7, 8]. A 2022 meta‐analysis concluded that CI may provide only short‐term relief in chronic cases [9].

Thermal radiofrequency ablation (RFA) is an emerging minimally invasive technique that provides sustained pain relief in chronic plantar heel pain by creating controlled thermal lesions in the sensory branches innervating the plantar fascia insertion, thereby interrupting nociceptive transmission, and potentially inducing structural remodeling of collagen fibers within the enthesis region [10, 11, 12]. Although both CI and RFA have been independently studied, direct comparisons are limited [13, 14]. Furthermore, no clinical study has yet systematically investigated the additive effect of combining RFA and CI in recalcitrant PF, despite their theoretically complementary mechanisms.

Musculoskeletal ultrasound (MSUS) is widely used for the diagnosis and follow‐up of plantar fasciitis (PF), allowing noninvasive measurement of plantar fascia thickness (PFT). A thickness greater than 4 mm is associated with increased symptom severity and disease chronicity [15, 16, 17].

In this retrospective matched‐cohort study, we compared the outcomes of RFA, CI, and their combination in patients with chronic plantar fasciitis. Pain [visual analog scale (VAS)], function [Foot Function Index (FFI) and Roles and Maudsley score (RMS)], plantar fascia thickness, and 12‐month relapse rates were evaluated. To our knowledge, this is one of the first studies to investigate combined RFA and CI therapy using both clinical and ultrasonographic outcomes in a well‐matched cohort. We hypothesized that the combined treatment would provide superior short‐ and long‐term outcomes compared with either.

## 2 Methods

### 2.1 Study Design

This retrospective single‐center observational study was conducted at the Algology Outpatient Clinic of Antalya Training and Research (blinded for reviewers) Hospital, a tertiary pain management center. A total of 156 patients with chronic plantar heel pain lasting more than six months and unresponsive to at least three months of conservative treatment, between January 2021 and June 2024, were included. The study was approved by the institutional ethics committee of Antalya Training and Research Hospital (Date: 29 May 2025, Decision No: 9/7) and conducted in accordance with the Declaration of Helsinki.

### 2.2 Setting, Screening, and Patient Selection

In routine clinical practice, patients underwent basic assessments involving a physical examination, MSUS, and VAS, FFI, and RMS scoring. Follow‐up assessments were performed at three, six, and 12 months using the same methods. All data were recorded in the hospital's electronic system and patient files. All analyses were performed using a complete case approach; patients with incomplete follow‐up or missing key outcome data were excluded from the final analysis.

In our clinic, the standard treatment approach for plantar fasciitis consists of a combination of ultrasound‐guided corticosteroid injection and radiofrequency ablation. However, in this retrospective study, some patients received only one of these modalities. Patients who did not undergo radiofrequency ablation either had medical contraindications to the procedure or did not meet the reimbursement criteria of the national health insurance system. Those who did not receive corticosteroid injections either had contraindications to corticosteroid therapy or declined this option. In cases without contraindications to either modality and upon obtaining patient consent, combined therapy was performed.

To enhance baseline comparability and reduce confounding, patients were matched across groups based on age (± 3 years), sex, BMI (± 2 kg/m^2^), and major comorbidities. However, despite adjustment efforts, the absence of randomization or propensity score matching means that residual confounding cannot be excluded. All patient data were anonymized, and the name of the institution was blinded for peer review compliance in accordance with ethical and editorial guidelines.

### 2.3 Interventional Protocols

**FIGURE 1:**
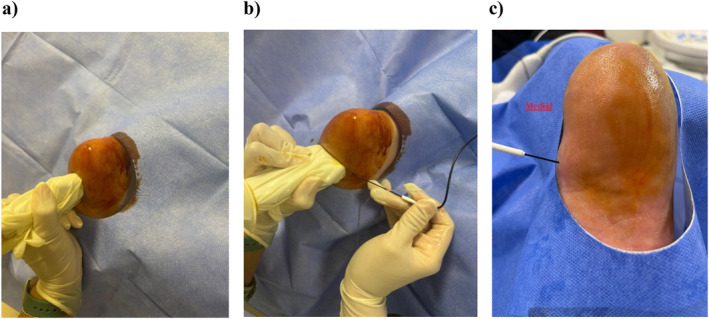
Technique of ultrasound‐guided radiofrequency ablation in plantar fasciitis. (a) Sterile preparation of the heel with the patient in the prone position and the foot dorsiflexed at 90° to optimize plantar fascia visualization. (b) Real‐time ultrasound‐guided in‐plane insertion of the RF cannula targeting the subfascial region adjacent to the medial calcaneal tubercle. (c) Final positioning of the cannula tip prior to ablation, with confirmation of proper placement on ultrasound imaging.

All procedures were performed under sterile conditions using real‐time ultrasound guidance with a 10–15 MHz linear probe. Patients lay in the prone position with the ankle dorsiflexed to 90°, and interventions were applied at the medial calcaneal tubercle using a longitudinal in‐plane approach, targeting the subfascial region. The needle entry site was infiltrated with a small amount of local anesthetic before needle advancement to minimize patient discomfort (Figure 1).

- RFA Group: A 22‐G RF cannula with a 5 mm active tip was inserted into the subfascial space. After confirming placement with sensory and motor stimulation, RFA was applied at 80°C for 90 s. No corticosteroids were administered.
- CI Group: A 22‐G spinal needle was used to inject 1 mL methylprednisolone acetate (40 mg/mL) and 1 mL 0.5% bupivacaine under ultrasound guidance. Proper dispersion was confirmed sonographically.
- Combined Group: The same RFA procedure was followed, immediately followed by the corticosteroid‐bupivacaine injection into the same area.

### 2.4 Ultrasound Evaluation

**FIGURE 2:**
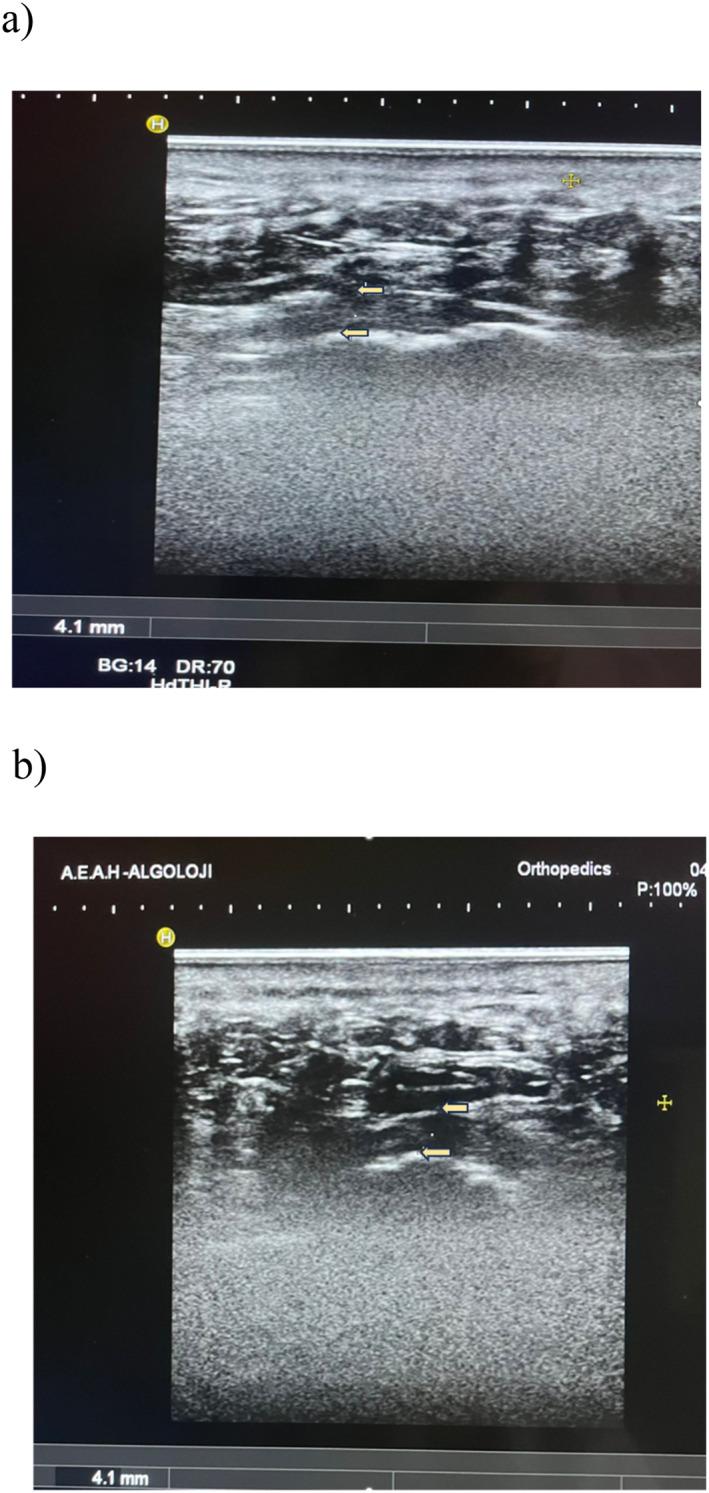
Ultrasonographic evaluation of plantar fascia thickness (PFT) in Patients with chronic plantar fasciopathy. (a) Longitudinal ultrasound image of the plantar fascia in a patient with recalcitrant plantar fasciitis, demonstrating thickening (4.1 mm) and hypoechoic changes near the calcaneal insertion. (b) Transverse ultrasound image of the plantar fascia in the same patient with recalcitrant plantar fasciitis, revealing persistent thickening (4.1 mm) at the calcaneal insertion.

All ultrasonographic procedures and interventions were performed by a single experienced pain specialist using a high‐frequency linear transducer (10–15 MHz) on a Hitachi HI VISION Preirus ultrasound system. Plantar fascia thickness was measured in the longitudinal plane at the medial calcaneal insertion with the ankle in neutral dorsiflexion (Figure 2). Measurements were recorded at baseline, 3 months, and 6 months. At the 12‐month follow‐up, measurement of plantar fascia thickness was performed only in patients who presented with clinical symptoms.

### 2.5 Clinical Outcome Measures

VAS, FFI, and RMS scores were recorded at baseline, 3 months, and 6 months. These were documented as part of standard clinical care and retrospectively reviewed. The Turkish validated version of the FFI was used [18]. VAS and RMS did not require cultural adaptation.

At the 12‐month follow‐up, all patients were systematically asked about the recurrence of heel pain during routine clinical visits. Those reporting symptoms underwent an additional musculoskeletal ultrasound examination to reassess plantar fascia thickness. Patients without clinical complaints did not undergo ultrasound imaging at this stage, in accordance with routine follow‐up procedures. All ultrasonographic evaluations, including those performed at the 12‐month visit, were conducted by the same experienced pain specialist (algologist), ensuring consistency. No additional clinical scoring tools or questionnaires were applied at the final follow‐up.

All clinical assessments, including baseline evaluations, follow‐up visits at 3 and 6 months, and the 12‐month relapse evaluations, were performed by the same senior orthopedic specialist. This ensured consistency in the application of clinical scoring systems (VAS, FFI, and RMS) and minimized interobserver variability. However, no blinding was performed for outcome assessors or participants due to the real‐world retrospective nature of the study, which we acknowledge as a limitation regarding potential observer bias.

### 2.6 Statistical Analysis

All analyses were performed using IBM SPSS Statistics for Windows, Version 25.0 (IBM Corp.). Continuous variables were assessed for normality using the Shapiro–Wilk test and presented as mean ± standard deviation (SD) or median (interquartile range, IQR) as appropriate. Categorical variables were reported as frequencies and percentages.

Between‐group comparisons were performed using one‐way ANOVA for normally distributed variables or the Kruskal–Wallis test otherwise. Chi‐squared tests were used for categorical comparisons. Within‐group changes over time (baseline, third month, and sixth month) in PFT, VAS, and FFI were analyzed using the Friedman test. The Wilcoxon signed‐rank test was applied for pre–post comparisons of RMS. Post hoc comparisons following significant Kruskal–Wallis results were made using Mann–Whitney *U* tests with Bonferroni correction. Statistical significance was defined as *p* < 0.05 (two‐tailed).

To evaluate clinical relevance, effect sizes were computed. Cohen's *d* was used for paired comparisons (VAS, FFI, and RMS), whereas eta squared (*η* ^2^) was reported for ANOVA results, where appropriate [19]. Although no formal a priori power analysis was performed due to the retrospective design, the sample size (*n* = 156) exceeded the estimated minimum required to detect a medium effect size (*d* = 0.5) with 80% power (*α* = 0.05) in a three‐group design [20].

## 3 Results

Baseline demographic and clinical characteristics were similar across the three groups (Table 1). The proportion of female patients was 48.1% in the RFA group (25/52), 52.0% in the CI group (26/50) and 53.7% in the combined group (29/54), with no significant differences. Mean age was 44.78 ± 6.33, 42.68 ± 5.24, and 43.46 ± 5.29 years and mean BMI was 31.02 ± 2.0, 30.88 ± 2.01, and 30.98 ± 2.00 kg/m^2^ in the RFA, CI, and combined groups, respectively, again without significant variation. Symptom duration was also comparable (8.12 ± 1.55, 8.34 ± 1.53, and 8.09 ± 1.75 months, respectively).

**TABLE 1:** Baseline demographic and clinical characteristics of patients in the radiofrequency ablation, corticosteroid injection, and combined therapy groups with statistical comparisons.

Variable	RFA group *N*: 52	CI group *N*: 50	Combined therapy group *N*: 54	*p*‐value
Female *n* (%)	25 (48.1%)	26 (52.0%)	29 (53.7%)	0.839
Age (years, mean ± SD)	44.79 ± 6.33	42.68 ± 5.24	43.46 ± 5.29	0.164
BMI (kg/m^2^, mean ± SD)	31.02 ± 2.00	30.88 ± 2.01	30.98 ± 2.00	0.937
Symptom duration (mo, mean ± SD)	8.12 ± 1.55	8.34 ± 1.53	8.09 ± 1.75	0.695

*Note:* Data are presented as number of patients (*n*) for categorical variables and as mean ± standard deviation (SD) for continuous variables. Group comparisons were performed using the chi‐squared test for categorical variables and one‐way analysis of variance (ANOVA) for continuous variables. No statistically significant differences were observed among the groups for any baseline characteristic.

Abbreviations: CI, corticosteroid injection; mo, months; RFA, radiofrequency ablation.

**FIGURE 3:**
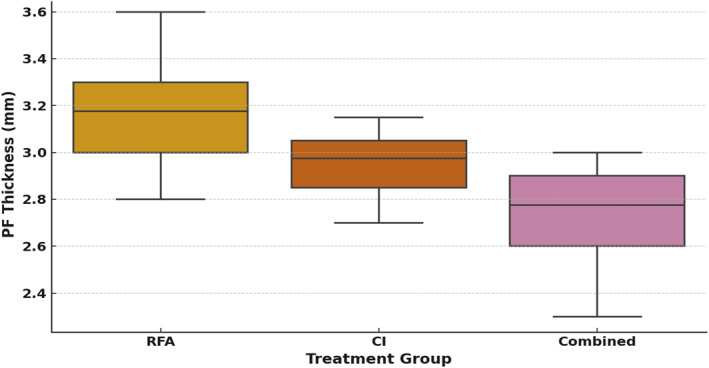
Plantar fascia thickness at 6 months by the treatment group. Box plots represent the median, interquartile range, and overall range of values for each intervention. The combined therapy group demonstrated the lowest median PF thickness compared to RFA and CI groups. CI, corticosteroid injection; CT, combined therapy; PF, plantar fascia; RFA, radiofrequency ablation.

Plantar fascia thickness decreased significantly in all groups over time (*p* < 0.001). Mean PFT reduced from 6.23 to 3.29 mm in RFA, from 6.17 to 2.95 mm in CI, and from 6.21 to 2.71 mm in the combined group. At 6 months, intergroup differences were significant, with the combined group showing the lowest PFT values, followed by CI, whereas RFA had the least reduction (Table 2, Figure 3).

**TABLE 2:** Within‐ and between‐group comparisons of plantar fascia thickness (mm) over time.

Group	Time point	Mean ± SD	Friedman *χ* ^2^ (df = 2)	*p*‐value	Pairwise comparisons (Wilcoxon and Bonferroni)	Between‐group (Kruskal–Wallis and Bonferroni)
RFA group	Baseline	6.23 ± 0.49	103.029	< 0.001	*Baseline vs 3m*: < 0.001; *Baseline vs 6m*: < 0.001; *3m vs 6m*: < 0.001	*Baseline*: NS; *3m*: CI > RFA, CI > combined (< 0.001); RFA vs combined: NS; *6m*: Combined > RFA, combined > CI (< 0.001); RFA vs CI: NS.
	3rd month	5.44 ± 0.48				
	6th month	3.29 ± 0.25				
CI group	Baseline	6.17 ± 0.53	97.578	< 0.001		
	3rd month	4.55 ± 0.89				
	6th month	2.95 ± 0.15				
Combined therapy group	Baseline	6.21 ± 0.59	103.600	< 0.001		
	3rd month	5.37 ± 0.45				
	6th month	2.71 ± 0.37				

*Note:* All groups showed significant reductions over time (Friedman test, *p* < 0.001). Post hoc analysis indicated greater improvement in the combined group at 6 months, whereas the CI group exhibited higher PFT values at 3 months compared to RFA and combined. Between‐group differences were analyzed with the Kruskal–Wallis test and Bonferroni‐corrected pairwise comparisons.

Abbreviations: CI: corticosteroid injection; NS: not significant; PFT: plantar fascia thickness; RFA: radiofrequency ablation.

VAS scores showed distinct patterns (Table 3). In CI, pain fell from 6.60 ± 1.16 at baseline to 3.30 ± 0.91 at 3 months but relapsed to 6.56 ± 1.30 at 6 months, returning to baseline levels. In RFA, VAS improved steadily from 6.73 ± 1.05 to 2.62 ± 0.57 at 3 months and 1.62 ± 0.63 at 6 months. In the combined group, VAS declined from 6.81 ± 1.01 to 2.41 ± 0.77 at 3 months and remained low at 1.83 ± 0.67 at 6 months. Between‐group differences were not significant at baseline but highly significant at 3 and 6 months (*p* < 0.001), with the largest mean reductions in RFA (−5.12) and combined therapy (−4.98), whereas CI showed almost no change (−0.04) (Table 3).

**TABLE 3:** Within‐ and between‐group comparisons of VAS scores over time.

Group	Time point	Mean ± SD	Friedman *χ* ^2^ (df = 2)	*p*‐value	Pairwise comparisons (Wilcoxon and Bonferroni)	Between‐group (Kruskal–Wallis and Bonferroni)
RFA group	Pretreatment	6.73 ± 1.05	96.525	< 0.001	*Baseline vs 3m*: < 0.001; *Baseline vs 6m*: < 0.001; *3m vs 6m*: < 0.001	*Baseline*: NS; *3m*: CI > RFA, CI > combined (< 0.001); RFA vs combined: NS; *6m*: CI > RFA, CI > combined (< 0.001); RFA vs combined: NS
	3rd month	2.62 ± 0.57				
	6th month	1.62 ± 0.63				
CI group	Pretreatment	6.60 ± 1.16	76.811	< 0.001	*Baseline vs 3m*: *p* < 0.001 *3m vs 6m*: *p* < 0.001 *Baseline vs 6m*: *p* = 0.864 (NS)	
	3rd month	3.30 ± 0.91				
	6th month	6.56 ± 1.30				
Combined therapy group	Pretreatment	6.81 ± 1.01	93.289	< 0.001	*Baseline vs 3m*: < 0.001; *Baseline vs 6m*: < 0.001; *3m vs 6m*: < 0.001	
	3rd month	2.41 ± 0.77				
	6th month	1.83 ± 0.67				

*Note:* Data are presented as mean ± SD. Within‐group changes (baseline, 3, and 6 months) were analyzed with the Friedman test and the Wilcoxon signed‐rank test (Bonferroni‐adjusted). Between‐group differences at 3 and 6 months were assessed using the Kruskal–Wallis test, with Mann–Whitney *U* tests (Bonferroni‐corrected) for post hoc analysis.

Abbreviations: CI, corticosteroid injection; NS: not significant; RFA, radiofrequency ablation.

Functional scores were also comparable at baseline (≈ 52 in all groups; Table 4). In RFA, FFI scores improved to 33.67 ± 2.66 at 3 months and 21.50 ± 3.43 at 6 months. In CI, scores declined to 32.04 ± 4.44 at 3 months but worsened to 46.62 ± 10.22 at 6 months, showing no significant difference from baseline. In the combined group, FFI fell to 28.76 ± 5.40 at 3 months and 17.57 ± 1.73 at 6 months, achieving the best results. At 6 months, both RFA and combined therapy were significantly superior to CI and the combined group also outperformed RFA.

**TABLE 4:** Within‐ and between‐group comparisons of FFI scores over time.

Group	Time point	Mean ± SD (mm)	Friedman *χ* ^2^ (df = 2)	*p*‐value	Pairwise comparisons (Wilcoxon and Bonferroni)	Between‐group comparisons (Kruskal–Wallis and Bonferroni)
RFA group	Pretreatment	52.38 ± 4.81	104.000	< 0.001	*Baseline vs 3m*: < 0.001; *Baseline vs 6m*: < 0.001; *3m vs 6m*: < 0.001	*3m*: Combined < CI (*p* = 0.013) and combined < RFA (*p* < 0.001); RFA vs CI: NS *6m*: Combined < RFA (*p* < 0.001) and combined < CI (*p* < 0.001); RFA < CI (*p* < 0.001)
	3rd month	33.67 ± 2.66				
	6th month	21.50 ± 3.43				
CI group	Pretreatment	52.40 ± 5.85	64.840	< 0.001		
	3rd month	32.04 ± 4.44				
	6th month	46.62 ± 10.22				
Combined therapy group	Pretreatment	52.31 ± 5.11	108.000	< 0.001		
	3rd month	28.76 ± 5.40				
	6th month	17.57 ± 1.73				

*Note:* Values are presented as mean ± standard deviation (SD). The Friedman test was used for within‐group comparisons across time points (baseline, third month, and sixth month). Pairwise comparisons within groups were performed using the Wilcoxon signed‐rank test with Bonferroni correction. Between‐group comparisons at each time point were assessed using the Kruskal–Wallis test, with post hoc pairwise comparisons adjusted using the Bonferroni method.

Abbreviation: NS: not significant.

RMS scores improved significantly in all groups (*p* < 0.001). In RFA, they decreased from 3.19 ± 0.66 to 1.40 ± 0.50; in CI, from 3.12 ± 0.66 to 2.44 ± 0.61; and in the combined group, from 3.15 ± 0.63 to 1.19 ± 0.39. Differences at 6 months were significant, with the best improvement in the combined group, followed by RFA (Table 5).

**TABLE 5:** Within‐ and between‐group comparisons of RMS scores.

Group	Pre‐treatment mean ± SD	6th month mean ± SD	Wilcoxon Z	Wilcoxon *p*‐value	K‐W H‐statistic (6M)	K‐W *p*‐value (6M)
RFA	3.19 ± 0.66	1.40 ± 0.50	−6.0	< 0.001	79.924	< 0.001
CI	3.12 ± 0.66	2.44 ± 0.61	−5.4	< 0.001		
Combined	3.15 ± 0.63	1.19 ± 0.39	−6.3	< 0.001		

*Note:* Values are presented as mean ± standard deviation (SD). Within‐group comparisons between baseline and sixth month were analyzed using the Wilcoxon signed‐rank test (Z, *p*‐value). Between‐group comparisons at the sixth month were performed using the Kruskal–Wallis test (H statistic and *p*‐value). *p* < 0.05 was considered statistically significant.

Abbreviations: CI, corticosteroid injection; RFA, radiofrequency ablation.

At 12 months, relapse occurred in 12/52 patients (23.08%) in RFA, 19/50 (38.00%) in CI, and 8/54 (14.81%) in the combined group. Overall group differences were significant (*p* = 0.022), with the excess recurrence in CI driving the effect. Pairwise analysis showed significantly fewer relapses in the combined group compared with CI (*p* = 0.008), whereas other comparisons were not significant (Table 6).

**TABLE 6:** Relapse rates at 12‐month follow‐up and pairwise comparisons.

Group	Relapse rate (% [*N*])	Pairwise *p*‐values (Fisher's exact)	Overall *χ* ^2^/*p*‐value
RFA	23.08 [12/52]	vs combined: *p* = 0.326	*χ* ^2^ = 7.597, *p* = 0.022
CI	38.00 [19/50]	vs RFA: *p* = 0.132	
Combined	14.81 [8/54]	vs CI: *p* = 0.008	

*Note:* Rates are expressed as the proportion of patients experiencing recurrence relative to the total number in each group. The overall comparison was performed using the chi‐squared test. Pairwise *p*‐values were obtained using Fisher's exact test.

Abbreviations: CI, corticosteroid injection; RFA, radiofrequency ablation.

Subgroup analysis revealed that patients who relapsed had higher VAS (4.18 ± 2.62 vs. 2.97 ± 2.31 and *p* = 0.006) and FFI scores (33.5 ± 15.5 vs. 26.4 ± 13.3 and *p* = 0.003) at 6 months, whereas PFT, BMI, and age were similar between relapse and nonrelapse groups (Table 7).

**TABLE 7:** Comparison of clinical and demographic variables between patients with and without relapse at 12‐month follow‐up.

Variable	Relapse mean ± SD	No relapse mean ± SD	U‐statistic	*p*‐value
VAS‐6M	4.18 ± 2.62	2.97 ± 2.31	2927.5	0.006
FFI‐6M (%)	33.49 ± 15.54	26.43 ± 13.34	3015.5	0.003
PFT‐6M (mm)	3.00 ± 0.32	2.97 ± 0.38	2309.5	0.907
BMI	30.69 ± 2.15	31.05 ± 1.94	2071.5	0.381
Age	43.67 ± 5.23	43.65 ± 5.83	2361.0	0.745

*Note:* Distribution of relapse events and relapse rates at the 12‐month follow‐up across treatment groups. Relapse was defined as the recurrence of heel pain after initial symptom improvement as reported during structured follow‐up visits. Relapse rates are calculated as the proportion of patients experiencing recurrence relative to the total number of patients in each group.

Abbreviations: CI, corticosteroid injection; RFA, radiofrequency ablation.

Effect sizes (Cohen's d) were calculated from paired preintervention and postintervention scores for VAS, FFI, and RMS. According to Cohen's criteria, values of 0.2, 0.5, 0.8, and ≥ 1.2 indicate small, medium, large, and very large effects, respectively.

## 4 Discussion

This retrospective study compared the clinical efficacy of CI, RFA, and their combination in the management of chronic plantar fasciitis. Although all three modalities provided statistically significant improvements in pain and function, the combination of RFA and CI consistently achieved the most favorable outcomes across clinical, functional, and morphological measures and was associated with the lowest relapse rate. To our knowledge, this is one of the first studies to assess their comparative effects within a single patient cohort. Importantly, baseline demographic and clinical characteristics, including age, sex, BMI, and symptom duration, were comparable across groups, reinforcing the validity of the between‐group comparisons.

The therapeutic effects of RFA and CI stem from fundamentally different mechanisms. Corticosteroids primarily exert anti‐inflammatory effects by reducing local cytokine release and perineural edema, which explains the rapid initial symptom relief observed in the CI group [9]. However, this effect was short‐lived, consistent with previous reports of relapse rates up to 40% by 6–12 months [7, 17]. In our study, the CI group showed a similar pattern, with symptom recurrence between months 3 and 6 and functional outcomes returning to baseline levels.

Conversely, RFA attenuates nociceptive transmission through thermal and neuroablative mechanisms. By targeting small unmyelinated C‐fibers and reducing aberrant neovascularization, RFA promotes longer‐term pain relief and may contribute to tissue remodeling [12, 14]. In our study, RFA was associated with sustained improvements in both pain and function as well as greater reductions in plantar fascia thickness compared with CI. These long‐term results are consistent with earlier studies on image‐guided radiofrequency ablation and support its role as an effective treatment for chronic plantar fasciitis [11, 21].

The most promising results were observed in the combination group. Patients receiving combined therapy experienced significant reductions in pain (VAS), marked improvements in function (FFI and RMS) and demonstrated the lowest relapse rate. At 6 months, the combined therapy group demonstrated the most pronounced decrease in plantar fascia thickness, surpassing both CI and RFA alone, suggesting that the integration of structural remodeling with rapid anti‐inflammatory effects yields superior outcomes. The large within‐group effect sizes confirmed robust clinical relevance, with RFA and combined therapy achieving very large effects, whereas CI produced only negligible‐to‐moderate benefits. Notably, the combined therapy group exhibited the lowest 12‐month relapse rate, which was significantly lower than that of the CI group, underscoring the durability of clinical benefits when RFA is integrated with corticosteroid injection. This synergy likely stems from corticosteroids rapidly suppressing inflammation in the acute phase, whereas RFA delivers long‐term benefit through collagen remodeling. This combined approach aligns with emerging evidence from other chronic tendinopathies, including lateral epicondylitis and Achilles tendinopathy, in which the integration of thermal, anti‐inflammatory, and mechanical modalities has been associated with superior clinical outcomes [22, 23].

Functional improvements were substantial, with marked gains in FFI and RMS scores, particularly in the combined therapy group. RMS scores improved significantly in all groups, with the largest effect observed in the combined cohort. Patients who experienced relapse had significantly higher VAS and FFI scores at 6 months, suggesting that a suboptimal mid‐term clinical response may be associated with an increased risk of recurrence. These findings are consistent with prior evidence demonstrating the limited durability of corticosteroid injections. For instance, Canyilmaz et al. reported that patients treated with steroid injections showed higher rates of symptom recurrence and required repeat interventions earlier compared with those receiving low‐dose radiotherapy, which provided more sustained analgesic effects [24].

An important observation was the association between symptomatic relapse and objective imaging findings. Although the 12‐month assessments were based on patient‐reported symptoms, all individuals reporting recurrence underwent ultrasound evaluation, which confirmed an increase in PFT. This protocol strengthens the validity of the relapse definition; however, its reliance on imaging triggered solely by the presence of symptoms is recognized as a methodological limitation. Procedural safety was favorable across all treatment modalities, with no adverse events, such as fascial rupture or infection observed, consistent with previous reports [9, 10, 11]. The use of ultrasound guidance likely contributed to procedural accuracy and minimized complications, especially during RFA.

### 4.1 Limitations

This study has certain limitations. Its retrospective and nonrandomized design carries a risk of selection bias, although treatment allocation followed predefined clinical criteria that likely minimized variability. Nonetheless, as treatment decisions were also influenced by patient preferences and reimbursement policies, the possibility of residual confounding cannot be excluded. No power analysis was conducted; however, post hoc effect sizes were calculated to support the robustness of the findings. Outcome assessors were not blinded, which may have influenced subjective measures, although ultrasonographic evaluations were standardized and performed by an experienced pain specialist. Finally, the single‐center design may limit generalizability, and multicenter prospective studies are needed to confirm these results.

### 4.2 Future Directions

Future studies should include prospective multicenter randomized trials with longer follow‐up to confirm these findings and enhance generalizability. Incorporating quality‐of‐life and cost‐effectiveness assessments, along with subgroup analyses, may help identify patients most likely to benefit from specific treatment combinations.

## 5 Conclusion

In conclusion, although both RFA and CI provided clinical benefits, combination therapy yielded the most pronounced and long‐term improvements in pain, function, and fascia morphology, along with the lowest relapse rate at 12 months. These findings highlight combined image‐guided treatment as the most effective and safe option for patients unresponsive to conservative care.

## Author Contributions

**Çile Aktan:** conceptualization, data curation, investigation, methodology, supervision, validation, writing – original draft, writing – review and editing. **Cemil Aktan:** formal analysis, funding acquisition, project administration, visualization, writing – review and editing.

## Ethics Statement

This study was approved by the Ethics Committee of Antalya Training and Research Hospital (Date: 29 May 2025, Decision No: 9/7) and conducted by the Declaration of Helsinki.

## Conflicts of Interest

The authors declare no conflicts of interest.

## Data Availability

The data that support the findings of this study are available from the corresponding author upon reasonable request.
